# Scalp eschar and neck lymphadenopathy after tick bite (SENLAT) caused by Bartonella henselae in Korea: a case report

**DOI:** 10.1186/s12879-020-4940-0

**Published:** 2020-03-12

**Authors:** Jun-Won Seo, Choon-Mee Kim, Na Ra Yun, Dong-Min Kim, Sung Soon Kim, Sangho Choi, Hyuk Chu

**Affiliations:** 1 Department of Internal Medicine, College of Medicine, Chosun University, 588 Seosuk-dong, Dong-gu, Gwangju, 61453 Republic of Korea; 2 Premedical Science, College of Medicine, Chosun University, Gwangju, Republic of Korea; 3 Division of Bacterial Disease Research, Center for Infectious Disease Research, Korea National Institute of Health, Chungcheongbuk-do, Korea

**Keywords:** Bartonella henselae, Spotted fever group Rickettsiosis, Tick-borne diseases, Lymph nodes

## Abstract

**Background:**

Tick-borne lymphadenopathy (TIBOLA) is an infectious disease, mainly caused by species from the spotted fever group rickettsiae and is characterized by enlarged lymph nodes following a tick bite. Among cases of TIBOLA, a case of scalp eschar and neck lymphadenopathy after tick bite (SENLAT) is diagnosed when an eschar is present on the scalp, accompanied by peripheral lymphadenopathy (LAP). Only a few cases of SENLAT caused by *Bartonella henselae* have been reported.

**Case presentation:**

A 58-year-old male sought medical advice while suffering from high fever and diarrhea. Three weeks before the visit, he had been hunting a water deer, and upon bringing the deer home discovered a tick on his scalp area. Symptoms occurred one week after hunting, and a lump was palpated on the right neck area 6 days after the onset of symptoms. Physical examination upon presentation confirmed an eschar-like lesion on the right scalp area, and cervical palpation revealed that the lymph nodes on the right side were non-painful and enlarged at 2.5 × 1.5 cm. Fine needle aspiration of the enlarged lymph nodes was performed, and results of nested PCR for the *Bartonella* internal transcribed spacer (ITS) confirmed *B. henselae* as the causative agent.

**Conclusion:**

With an isolated case of SENLAT and a confirmation of *B. henselae* in Korea, it is pertinent to raise awareness to physicians in other Asian countries that *B. henselae* could be a causative agent for SENLAT.

## Background

*Bartonella henselae* is a gram-negative, facultative, intracellular bacteria that can cause various diseases, including lymphadenopathy, bacteremia, bacillary angiomatosis, and bacillary peliosis [1]. One of the typical diseases from *B. henselae* is cat scratch disease. People usually contract the disease from cats infected by *B. henselae*, but cases from flea or tick bites have been reported [2]. The infection is asymptomatic in cats, but for humans, it can result in symptoms or signs such as lymphadenopathy, red papules, fever, headache, malaise, and sometimes, in adults, fever of unknown origin (FUO) [3].

Tick-borne lymphadenopathy (TIBOLA) is an infectious disease, mainly caused by species from the spotted fever group rickettsiae (e.g. *Rickettsia slovaca, Rickettsia raoultii*) and is characterized by enlarged lymph nodes following a tick bite. Scalp eschar and neck lymphadenopathy after tick bite (SENLAT) occurs after a bite from a tick and key clinical features occur at the surface of the scalp and cervical lymph nodes. Therefore, we consider the TIBOLA case with eschar on the scalp as SENLAT [4]. There are some cases of SENLAT caused by *B. henselae* in other country [5], but there are no such case reports in South Korea, except for some other clinical syndromes [1, 6–8]. This study reports a first case of SENLAT in which *B. henselae* was confirmed as the etiologic agent in Korea.

## Case presentation

**Fig. 1:**
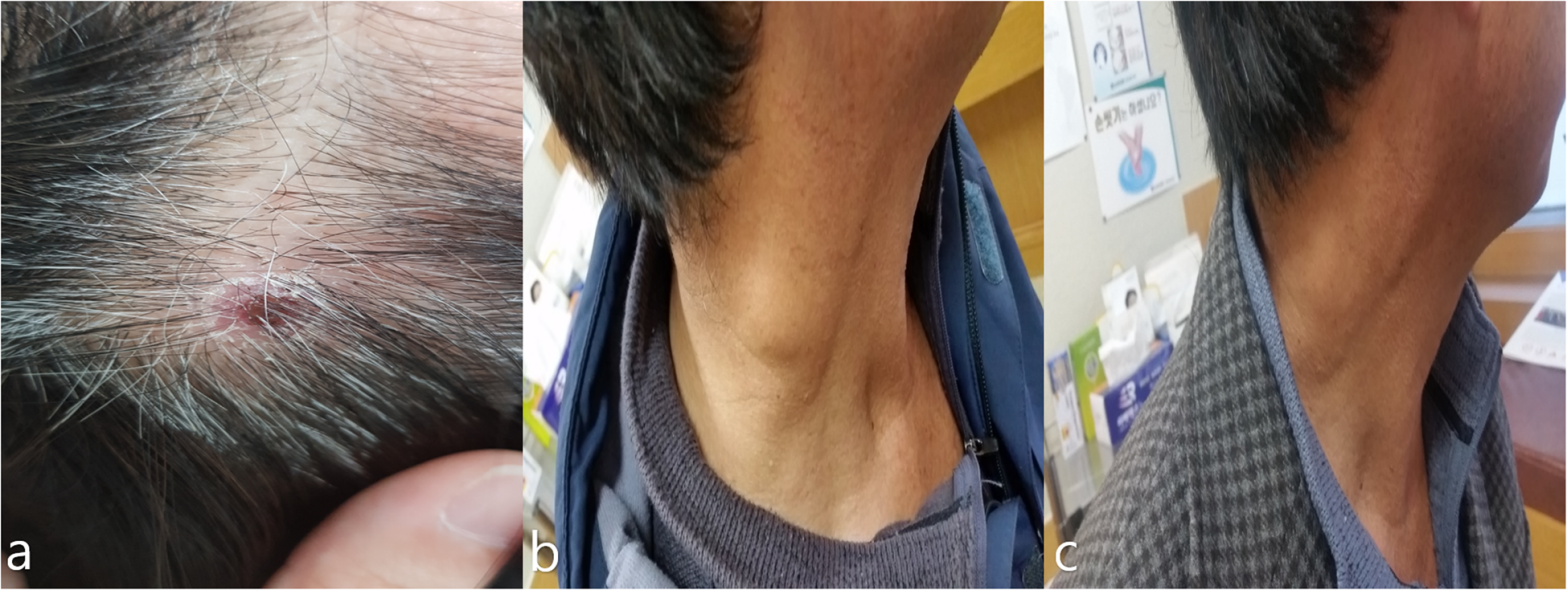
A photograph of the eschar on the scalp and right cervical area of a 58-year-old male patient with a confirmed diagnosis of Bartonella henselae, and a cytology report from fine needle aspiration of an enlarged cervical lymph node. **a** Eschar on the scalp at the first visit to the outpatient clinic. **b** Right cervical lymphadenopathy on the first visit to the outpatient clinic. **c** A photograph showing a marked reduction of size in the right cervical lymphadenopathy 10 days later

The patient was a 58-year-old male, who brought home a water deer (*Hydropotes inermis argyropus*) from Muan-gun, Jeollanam-do, Korea., he had hunted a week prior to his presentation. His symptoms of high fever, diarrhea, and indigestion developed after the hunting incident, and his right cervical lymph nodes suddenly became swollen 6 days following the onset of fever, which prompted him to visit the infectious diseases outpatient clinic at the Chosun University Hospital. The day after carrying the water deer home, he found a tick on his scalp, but had quickly removed and discarded it. He denied contact with cats or dogs as well as flea. On physical examination, he had a high fever of 39 °C, an eschar-like lesion was found on his right scalp area (Fig. 1a), and on palpation, non-painful peripheral lymphadenopathy (LAP) of 2.5 × 1.5 cm in size was identified in the right cervical region (Fig. 1b).

Blood test and fine needle aspiration were performed on the day of first visit. Laboratory investigations revealed a white blood cell count of 6.04 × 10^3^/uL, hemoglobin level of 17.2 g/dL, and platelet count of 313 × 10^3^/uL on routine complete blood count. Serum biochemistry revealed the following: total protein concentration, 6.96 g/dL; albumin, 4.44 g/dL; blood urea nitrogen, 18.0 mg/dL; bilirubin, 0.55 mg/dL; alkaline phosphatase, 52 U/L; and creatinine, 1.00 mg/dL (all were within normal limits). Aspartate aminotransferase (AST) of 29.5 U/L was within normal limits, but alanine aminotransferase (ALT; 45.8 U/L) as well as lactate dehydrogenase (LDH; 421 U/L) were mildly elevated. To identify the cause of LAP, fine needle aspiration (FNA) was performed on the enlarged lymph nodes of the neck.

Cytology from the FNA demonstrated a granuloma with an unclear boundary comprised of epithelioid cells along with giant cells and some lymphocytes.

**Fig. 2:**
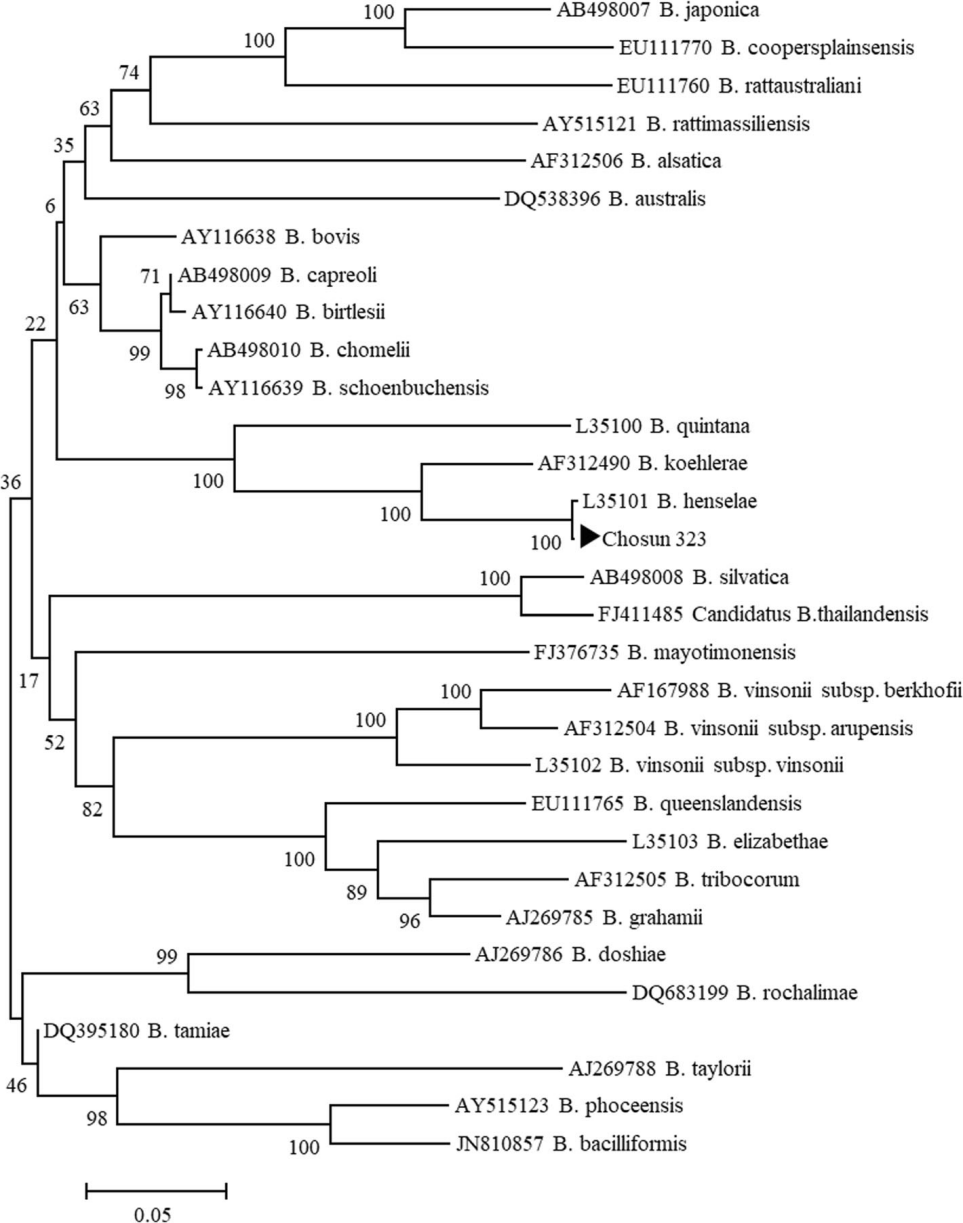
A phylogenetic tree based on Bartonella internal transcribed spacer (ITS) sequences from GenBank

DNA was extracted from the buffy coat of the patient’s blood and from the lymph node aspirate using a QIAamp Blood Mini kit (QIAGEN, Germantown, MD). The results of the genetic detection were all negative when the 56-kDa gene from *O. tsutsugamushi* and the *ompA* gene were targeted by nested PCR for rickettsial detection [9]. Nested PCR on the *Bartonella* internal transcribed spacer (ITS) [10], using blood and lymph node samples, and by using *B. elizabethae* as a positive control, revealed a positive band by electrophoresis in only the lymph node aspirate. Sequencing of the sample was therefore requested at SolGent (Daejeon, Korea). The query output of BLASTN (NCBI) demonstrated a 100% identical sequence (703/703 bp) to the *B. henselae* strain BM1374165 (accession no. HG969191) previously identified in human blood (Fig. 2).

Indirect immunofluorescent antibody assay (IFA) against *B. henselae* were conducted at at the Korea Centers for Disease Control and Prevention. The sera were examined with commercially available slides for Bartonella-IFA IgG and IgM assay (Focus Diagnostics, Cypress, CA, USA). The kit for detecting IgM and IgG antibodies utilizing Vero cells infected with either *B. henselae* or *B. quintana* was used according to the manufacturer’s instructions. Diagnostic criteria are determined to be *Bartonella* positive when endpoint titer of IgG ≥1:64 or IgM ≥1:20. The IFA IgM antibody titer against *B. hensealse* was < 1:20 at both first visit and follow up. The IFA IgG antibody titer against *B. hensealse* was < 1:16 at first visit (2015.12.07), and 1:16 after follow up (2016.01.04). He was treated by doxycycline for first 5 days and then with azithromycin for 5 days. Ten days later, the LAP resolved (Fig. 1c). Serological tests of *Orientia* and other *Rickettsia* species were performed together and the results were all negative.

## Discussion and conclusion

The incidence of disease caused by *B. henselae* has been previously reported, especially in association with contact with cats or dogs. The main route of infection is thought to be from scratching the site of a cat bite or a flea bite. The prevalence of *Bartonella* infection in Korea, identified by PCR, is estimated to be 0–44.1% [11, 12] in animals, and 0–19.1% [11, 13, 14] in arthropod vectors, but case reports of *Bartonella* infections in Korea have been rare. Just a few studies have been published about case of *B. henselae* infection in South Korea [1, 7, 8]. Kwon et al. reported 5 cases in which *B. henselae* was identified from cultures of blood or bone marrow [6]. However, there have been no reports of SENLAT by *B. henselae* in Korea.

TIBOLA commonly occurs in women and young people and has been reported in European countries such as France, Spain, and Hungary, particularly in cold seasons. *Rickettsia slovaca* is known to be the most commonly confirmed etiologic agent of TIBOLA, and the most frequently identified vector is *Dermacentor marginatus* [15]. The scalp area is recognized as the most common site for tick bites. One possible explanation for this is that *Dermacentor* ticks can stick to long hair, which plays a role as a shelter. Among TIBOLA entities, disease entity with both the eschar in the scalp and the neck lymphadenopathy are recognized as a new clinical entity named by SENLAT [8], as in our case. SENLAT has characteristic epidemiological findings that occur frequently in females and young children and are seasonal bimodality (spring and autumn) [4]. Although *Rickettsia slovaca* is the most common pathogen in this syndrome, other several agents like *Rickettsia raoultii*, *Rickettisa sibirica* subsp. *mongolitimonae, Coxiella burnetii, Borrelia burgdorferi*, and *Candidatus* Rickettsia rioja are also known as etiological pathogens [4]. The patient in our case had an eschar lesion on his right scalp, and a superficial enlarged right cervical lymph node, consistent with SENLAT. The result of the nested PCR, using a sample from the enlarged node, was positive for *Bartonella* species in a genus-specific ITS gene, and the sequencing results confirmed *Bartonella henselae* to be the cause of infection. And then, based on previous several literature showing that water deer living in Korea serve as a reservoirs of *Bartonella* species [16, 17], we believed that *B. henselae* identified in our patient originated from a water deer contacted prior to our hospital visit. In our case, the tick residing on the water deer may have bitten the patient, and hence infected him with *B. henselae*. As the patient had discarded the tick, we could not investigate the role of the tick as a vector. *Dermacentor* ticks are, however, known to be absent in Korea [18]. Cotte et al. showed in their experimental study that potential transmission of *B. henselae* is possible with *Ixodes ricinus* ticks [19]. Therefore, one cannot exclude a possibility that SENLAT could have been caused by *Ixodes nipponensis*, which is frequently observed in Korea [20]. In addition, the water deer may be the source of the tick, but it is not clear, and the natural environment may have been the tick source. However, further study is needed to confirm this.

Angelakis et al. have reported SENLAT caused by *B. henselae* following a tick bite [5]. These three patients who are similar to our patient, but there is one big difference. That is, they are proven to be infected by *B.henselae* through PCR test with eschar tissue or tick specimen, but we have demonstrated SENLAT by *B.henselae* with PCR tests using neck lymph node tissue. All 3 of their examples occurred in the colder months in Europe, and the authors suggested that *Dermacentor* ticks are most active during these periods. The occurrence of SENLAT has mainly been reported in Europe. However, no cases of SENLAT have been reported in Asia.

Difficulties in culturing *B. henselae* from pus aspirates and lymph node biopsy specimens of patients with cat scratch disease have been reported [21], and very low levels of sensitivity in serologic and PCR tests have been found against *B. henselae* infection. For example, among the 18 patients with a confirmed diagnosis of cat scratch disease, only 3 were noted to have positive PCR results, and the cycle thresholds were reported to be average of 38 (range: 37.7–39.3) [22]. Our patient also showed a positive PCR result for *B. henselae* with lymph node aspirate, but not with a blood sample. IFA antibody test for *B. henselae* was also negative, presumably due to the low sensitivity of the IFA IgG antibody test [23].

In conclusion, this case report demonstrated a case of SENLAT in which the patient had ipsilateral LAP and a scalp eschar, with a confirmed diagnosis of *B. henselae* infection from PCR of aspirate from the affected lymph node. This study should raise awareness in clinicians that, in addition to *Rickettsia* species, *B. henselae* may be a causative agent of TIBOLA or SENLAT.

## Data Availability

All the information supporting our conclusions and relevant references are included in the manuscript. There are no datasets related to this case report.

## Abbreviations

ALT: Alanine aminotransferase
AST: Asparate aminotransferase
FNA: Fine needle aspiration
FUO: Fever of unknown origin
IFA: Immunofluorescence assay
ITS: Internal transcribed spacer
LAP: Lymphadenopathy
LDH: Lactate dehydrogenase
PCR: Polymerase chain reaction
SENLAT: Scalp eschar and neck lymphadenopathy after a tick bite;
TIBOLA: Tick-borne lymphadenopathy
